# Supplementary material for: Anxiety and depression in Guatemala: Sociodemographic characteristics and service access
Source: PLoS One. 2022 Aug 12;17(8):e0272780. doi: 10.1371/journal.pone.0272780 (PMC9374225; doi:10.1371/journal.pone.0272780)
Supplement: Attachment — Submitted filename: Response to Reviewers.docx [file pone.0272780.s001.docx]

Dear Editor,

We thank you for the opportunity to resubmit our revised manuscript “Anxiety and depression in Guatemala: sociodemographic characteristics and service access”.

We also thank the reviewers for their thorough and helpful comments which we have responded to and which have strengthened the manuscript.

We have responded below to the ‘General Comments’ from PLOS ONE as well as to each of the reviewers and have made the revisions accordingly in the manuscript.

We look forward to hearing from you.

Yours faithfully,

Jonathan Naber

-----

**General comments: Journal requirements:**

1. Please ensure that your manuscript meets PLOS ONE's style requirements, including those for file naming. The PLOS ONE style templates can be found at

https://journals.plos.org/plosone/s/file?id=wjVg/PLOSOne_formatting_sample_main_body.pdf and

<https://journals.plos.org/plosone/s/file?id=ba62/PLOSOne_formatting_sample_title_authors_affiliations.pdf>.

RESPONSE:

The article does meet the PLOS ONE style requirements to the best of our awareness. We will ensure to obey the file naming requirements for all files of the re-submission.

2. We note that the grant information you provided in the ‘Funding Information’ and ‘Financial Disclosure’ sections do not match. When you resubmit, please ensure that you provide the correct grant numbers for the awards you received for your study in the ‘Funding Information’ section.

RESPONSE:

Thank you for drawing this to our attention. We will ensure the grant numbers match between the Funding Information and Financial Disclosure sections in the re-submission.

3. Thank you for stating the following in the Acknowledgments Section of your manuscript:“CONADI, CBM, and UNICEF for financing this study.”

We note that you have provided funding information that is not currently declared in your Funding Statement. However, funding information should not appear in the Acknowledgments section or other areas of your manuscript. We will only publish funding information present in the Funding Statement section of the online submission form.

Please remove any funding-related text from the manuscript and let us know how you would like to update your Funding Statement. Currently, your Funding Statement reads as follows:

“The Guatemala survey was funded by CBM Latin America and CONADI (the National Council on Disability), in a grant to Dr. Sarah Polack. No specific funding was received for these analyses of the data.”

Please include your amended statements within your cover letter; we will change the online submission form on your behalf.

RESPONSE:

Thank you for drawing this to our attention. In the edited manuscript, we deleted the funding information from the acknowledgements section.

The modified Funding Statement should read: ‘The Guatemala survey was funded by CBM Latin America, CONADI (the National Council on Disability), and UNICEF, in grants to Dr. Sarah Polack. No specific funding was received for these analyses of the data.

4. In your Data Availability statement, you have not specified where the minimal data set underlying the results described in your manuscript can be found. PLOS defines a study's minimal data set as the underlying data used to reach the conclusions drawn in the manuscript and any additional data required to replicate the reported study findings in their entirety. All PLOS journals require that the minimal data set be made fully available. For more information about our data policy, please see [http://journals.plos.org/plosone/s/data-availability](http://protect-eu.mimecast.com/s/rgy_Cmy54uR256GHDyyHX?domain=journals.plos.org).

Upon re-submitting your revised manuscript, please upload your study’s minimal underlying data set as either Supporting Information files or to a stable, public repository and include the relevant URLs, DOIs, or accession numbers within your revised cover letter. For a list of acceptable repositories, please see [http://journals.plos.org/plosone/s/data-availability#loc-recommended-repositories](http://protect-eu.mimecast.com/s/HkJiCnO14c65GxQhpZNQE?domain=journals.plos.org).

Any potentially identifying patient information must be fully anonymized.

Important: If there are ethical or legal restrictions to sharing your data publicly, please explain these restrictions in detail. Please see our guidelines for more information on what we consider unacceptable restrictions to publicly sharing data: [http://journals.plos.org/plosone/s/data-availability#loc-unacceptable-data-access-restrictions](http://protect-eu.mimecast.com/s/buYsCoy14uK4X8Ji7tBL_?domain=journals.plos.org).

Note that it is not acceptable for the authors to be the sole named individuals responsible for ensuring data access.

We will update your Data Availability statement to reflect the information you provide in your cover letter.

5. We note that you have indicated that data from this study are available upon request. PLOS only allows data to be available upon request if there are legal or ethical restrictions on sharing data publicly. For more information on unacceptable data access restrictions, please see [http://journals.plos.org/plosone/s/data-availability#loc-unacceptable-data-access-restrictions](http://protect-eu.mimecast.com/s/buYsCoy14uK4X8Ji7tBL_?domain=journals.plos.org).

In your revised cover letter, please address the following prompts:

a) If there are ethical or legal restrictions on sharing a de-identified data set, please explain them in detail (e.g., data contain potentially sensitive information, data are owned by a third-party organization, etc.) and who has imposed them (e.g., an ethics committee). Please also provide contact information for a data access committee, ethics committee, or other institutional body to which data requests may be sent.

b) If there are no restrictions, please upload the minimal anonymized data set necessary to replicate your study findings as either Supporting Information files or to a stable, public repository and provide us with the relevant URLs, DOIs, or accession numbers. For a list of acceptable repositories, please see [http://journals.plos.org/plosone/s/data-availability#loc-recommended-repositories](http://protect-eu.mimecast.com/s/HkJiCnO14c65GxQhpZNQE?domain=journals.plos.org).

We will update your Data Availability statement on your behalf to reflect the information you provide.

RESPONSE:

There are legal/ethical restrictions because we did not obtain consent from study participants for sharing the database publically/outside of the research team. We can however provide the details of contact (non-author of the study) for where requests for further information about the data can be sent as follows: [Jyoti.Shah@lshtm](about:blank). We hope this is acceptable.

6. We note that you have included the phrase “data not shown” in your manuscript. Unfortunately, this does not meet our data sharing requirements. PLOS does not permit references to inaccessible data. We require that authors provide all relevant data within the paper, Supporting Information files, or in an acceptable, public repository. Please add a citation to support this phrase or upload the data that corresponds with these findings to a stable repository (such as Figshare or Dryad) and provide and URLs, DOIs, or accession numbers that may be used to access these data. Or, if the data are not a core part of the research being presented in your study, we ask that you remove the phrase that refers to these data.

RESPONSE:

We have now removed this “data not shown” as the estimate is actually included in the manuscript

7. Your ethics statement should only appear in the Methods section of your manuscript. If your ethics statement is written in any section besides the Methods, please delete it from any other section.

RESPONSE:

The ethics statement does not appear in any section outside of the Methods section to the best of our awareness.

-----

**Reviewers’ Comments**

Reviewer #1:

This article contribute to the lack of epidemiological data on mental health disorders in Guatemala. Studies like this are crucial to improve people’s life through the needed policy changes at the regional and national level.
It is important to notice that the data collection took place in 2016, reducing is validity and direct implications. However, there is a high need of mental health data from Guatemala and other neighboring countries that make the study significant.

RESPONSE:

We thank the reviewer for their reflections and very helpful feedback.

Another major concern is the assessment selected. Even though it is promising, The Washington Group extended set on functioning (WG-ES) is s recent tool. I recommend a more extended description of the tool and justification of its use. Also to include any available data on psychometric characteristics of the survey along with validity and comparison with other more commonly used assessments of mental health disorders.
The discussion does not go deep on the inconsistent findings. More reflection and potential hypothesis should be presented to better discuss the interesting results.

RESPONSE:

We have now included more detail about the WG-ES tool as well as references relating to its development. Some aspects of validity and reliability have been assessed in other settings, which we now mention and reference [Page 6 Paragraph 2]. We have also included a more detailed discussion of the limitations of this tool in the discussion specifically in relation to assessing anxiety and depression [Page 25 Paragraph 2].

Page 4: While this study generated important data for Guatemala, it only included adults between the ages of 18 and 65, effectively excluding a large part of the population.4
Population from 18-65 does not exclude a large part of the general population. Please be more accurate with the statements. Maybe you can say excluding older adults which represent and children X % of the population.

RESPONSE:

Thank you for this suggestion. According to the 2018 census, 44.7% of the population are <19 years and 5.6% are older than 65 years. As suggested, we have therefore added that this ‘effectively excludes half the total population’.

Methods:
Page 6: Self-reported functional difficulties
This is a very vague term. Is this the formal term used by the questionnaire? If so, please explain. Starting the case definition with this term reduces the article credibility.

RESPONSE:

Functioning/functional difficulties is the term used by the questionnaire developers and ‘Functioning’ is included in the title of the tool. We have now provided more details about the tool and the approach it uses to measure self-reported functional difficulties in [Page 7 Paragraph 2].

Page 7: Variables description.
More information about the variables is needed. From the main outcomes to the covariates. What combined depression and anxiety means? Explain outcome variables and scoring in detail to increase test validity (and author’s credibility).

RESPONSE:

- We have now provided more detail on the WG questions on anxiety and depression and references relating to their development and testing. The WG tool developers refer to these together as ‘affect’. However, in response to concerns raised by reviewer 2, we have now removed the term ‘affect’ and state the terms anxiety and/or depression. We combined anxiety and depression for the outcome measure purposes of comparing people with mental health difficulties, compared to people with functional difficulties in the other WG domains (sensory, physical, communication and cognitive). We have re-worked the data analysis section to make this clearer [Page 10 Paragraph 3].
- The limitations of using the WG questions to assess anxiety and depression are also discussed in more detail in the limitations section [Page 25 Paragraph 2].
- We have also included more information on collection of data on the co-variates and section newly titled as ‘co-variates’ [Page 8].

It would be useful to have more information about the survey questions. For example, number of items, examples of questions related to access to health services or livelihood, ect. More information about the variables is needed.

RESPONSE:

Thank you for this comment. We have now added details on the questions that were asked relating to education, livelihood and health [Page 8 Paragraph 3].

Page 8: The WG instrument was translated into the four leading non-Spanish languages of Guatemala - K’iche’, Kaqchikel, Mam, and Q’eqchi’.
Was this translation process similar to the English to Spanish or was it translated directly from the Spanish translation? Did you calculate internal validity of the tool in the different languages? Any information about its psychometric characteristics would be useful.

RESPONSE:

These translations were made from Spanish into the four non-Spanish languages; they were forward translated and back-translated to check concepts were retained. The questionnaires were pilot tested in each language to assess appropriateness, comprehension and equivalence, with adaptations made accordingly. [Page 9 Paragraph 2] We did not conduct formal analysis of psychometric properties of the WG tool. However, some evidence of convergent validity is indicated because i) distribution of participants reporting none, some and a lot of difficulty aligns with previous studies in other settings ii) the proportion reporting difficulty with different functional domains increased significantly with age, which would be expected and is shown consistently in other studies.

Page 8: “Two weeks of mop-up were conducted at the end of the survey in clusters with low response rates or incomplete data, primarily in the Central and Northwest regions.” In what this consisted. I guess that if you mention it is because it is enough important to be better explained. Please provide further details.

RESPONSE:

‘Mop up’ involved revisiting clusters with low response rates/incomplete data. The intention was to maximise response rate and data completion. We have edited this text to make this clearer.

Results:
The age distribution is very limited. The sample is not evenly distributed at all after all the sampling described in the methods. From the 13073 total sample, only 2035 are above 50 years old. Is it representative of the Guatemaltecan population (15% of people are >50).

The age and sex distribution of the study sample were broadly similar to that of the national population based on the 2018 national census, suggesting our sample is representative of the Guatemalan population in terms of age distribution. We have now included a table (Table 2) and comment in the text [Page 12] to show this.

Page 14: However, of 385 cases with impaired affect, only 25 (6%) had received medication for depression or anxiety in the past 12 months.
My recommendation is to avoid any judgement in the results section and leave them for the discussion.

RESPONSE:

Thank you for this helpful suggestion, we have now edited accordingly.

Discussion:

1. Page 19: There is an increased need for mental health services targeting the 50+ segment of the population.
   What percentage of the total population it represents? What kind of disorders they may be facing. What are the implications or future studies related ot that?
2. In general, results could be compared with other similar studies/surveys from neighboring countries or at least other Latino countries.

RESPONSE:

Thank you for these helpful suggestions. According to the 2018 National Census, 14.7% of the population is 50+ years of age - we have now added this information to the text. We also added further information about how this finding in Guatemala differs from the results of the 2019 Global Burden of Disease Study. [Page 22 Paragraph 1].

Page 20: it also found that literacy was not significantly associated with impaired affect. However, ENSM found that people who ‘’Know how to read and write’’ had a higher likelihood of affective and anxiety disorders.4 This disagreement calls for further research on the relationship between low literacy and impaired affect, considering a review by Maughan et al. which found some co-occurrence of reading failure with anxiety and depression.24
It would be interesting to know, if available, how many people had a low literacy level from the survey and how the informed consent was done on those cases. If you have this data, it would be useful in order to increase the information about the results.

RESPONSE:

Overall, 23% (n=2760) of the survey population reported that they ‘cannot read at all’. These numbers are provided in Table 2 (*now 3). In terms of consent, information sheets were read out loud to all participants and for people unable to write, thumb-printed consent was obtained. This information has now been added to the ethics section [Page 10 Paragraph 2]

Page 20: We found no significant association between ethnic group and impaired affect in this study. This was unexpected given that ENSM found greatly-increased odds of having depression or post-traumatic stress disorder (PTSD) in indigenous people who had been exposed to violence when compared to non-indigeous people who had also been exposed to violence.27 Another study, however, found that “socioeconomic and health-related variables” greatly confounded the relationship between ethnic group and impaired affect.25,28 We found no association between SES and impaired affect in this study. This was surprising given the expanding evidence on the inverse relationship between SES and depression.29 Further research on ethnicity and socioeconomic status is needed to clarify these unexpected results.
These are indeed very odd results. Please expand on potential explanations and what the literature from other countries says on this respect as it questions the validity of the study (test validity). For example, is there any literature about resilient factors among indigenous population that may explain that 9 years later this subgroup is doing better? At least, you should mention the limitation of the lack of use of this survey and culturally adapted tools for these subgroups as this might be the reason for not finding any differences.

RESPONSE:

We did find that anxiety and/or depression was more common among females, people with lower levels of education and those with other functional difficulties. These findings align with trends expected based on previous literature which gives some indication of test validity). It is possible, therefore, that the lack of association with SES may actually reflect limitations with the way SES was measured. We have now added further information about potential limitations of the SES measurement approach which may have contributed to the lack of association observed [Page 23 Paragraph 3]

Page 21: Community health workers staffing these posts should be trained and equipped to provide mental health services in addition to physical health services to address their co-occurrence.
If the authors decide to recommend interventions, more justifications and detail should be provided. This sentence should at least be referenced. See “Alegría M, et al. Effectiveness of a Disability Preventive Intervention for Minority and Immigrant Elders: The Positive Minds-Strong Bodies Randomized Clinical Trial. Am J Geriatr Psychiatry. 2019 Dec;27(12):1299-1313. doi: 10.1016/j.jagp.2019.08.008. Epub 2019 Aug 13. PMID: 31494015; PMCID: PMC6842701.” For a useful reference.

RESPONSE:

Thank you for helpfully sharing this reference. We have now expanded this section to highlight and reference literature about potential benefits of community level mental health and task sharing interventions, in order to further justify our recommendation [Page 25 Paragraph 1]

Page 21: or from ‘.... cultural differences in interpretation of the tools….’
I don’t understand what this sentence means in the middle of the discussion. Is this a qualitative quote from an investigator? This sentence lacks scientific rigor. Please review and correct for accuracy and add references to discuss this important aspect of the study. This is the most important limitation.

RESPONSE:

We have now edited and expanded the limitations section to include a much more detailed discussion of the limitations relating to the approach we used to assess anxiety and depression [Page 25 Paragraph 2]

Page 22: Conclusions
Some indication to the policy level needed changes should be included here. How these results can serve the policy makers to improve the quality of life and services among people with impaired affect?

RESPONSE: Thank you for this suggestion. We have now included more detail on recommendations and references in the Discussion section..

Reviewer #2:

The study deals with a relevant topic and is based on national epidemiological data. However, there are several issues that are unclear and make it difficult to understand the theoretical framework, methods, results and conclusions.

1. The term “impaired affect” is inadequate to designate people with anxiety and depressive symptoms. This issue is central to the study, as the authors assume this concept, including the terms anxiety and depression in the title, when in fact there is no formal assessment of these symptoms/disorders.

RESPONSE

Thank you for this helpful feedback. ‘Affect’ is the term used by the developers of the WG tools. However, we understand the reviewers' concerns with this and that is potentially misleading. Therefore, we have taken out ‘impaired affect’ and instead use the ‘anxiety and/or depression’ throughout the manuscript. We have also included more detailed reflections of the possible limitations of the approach we used to assess anxiety and depression in the limitations section of the discussion. [Page 25 Paragraph 2]

The correct name of the instrument used (CIDI) is not Compact International Diagnostic Interview, but Composite International Diagnostic Interview.

RESPONSE:

Thank you for noting this error. This is now corrected.

The entire theoretical framework of the study is based on the lack of studies/ epidemiological data on mental health in Guatemala. The authors review these studies and describe the prevalence of mental disorders, showing that anxiety/somatoform and mood disorders are the most prevalent. However, there is no description of studies that have evaluated impaired affect and we cannot infer that these can be automatically translated into anxiety and mood disorders.

Thus, there is an important gap in the theoretical framework, which does not allow us to support the assertion that “The substantial gap in mental health spending and treatment in Guatemala suggests that people with impaired affect may face heightened burdens compared to people with other disabilities or with no disabilities.”

RESPONSE:

Thank you for this helpful comment. ‘Affect’ is the term used by the developers of the WG tools. However, we understand the reviewers' concerns with this and that is potentially misleading. Therefore, we have taken out ‘impaired affect’ and instead use the terms anxiety and/or depression throughout the manuscript. We have also included more detailed reflections of the possible limitations of the approach we used to assess anxiety and depression in the limitations section of the discussion. [Page 25 Paragraph 2]

The instrument/questionnaire used to define the case should be described in greater detail. How was this questionnaire constructed? It would be important to include references to the instrument.

RESPONSE:

Thank you. We have now included more details about the tools and references related to its development and testing.

The authors describe that both anxiety and depression were assessed using two questions, which included frequency (daily and a lot) for their classification. It would be important for the authors to provide references for the criteria adopted for the definition of impaired affect considering these questions. Which studies have used these criteria?

RESPONSE:

We adopted the criteria recommended by the tools developers (WG) and as used in previous studies; references for both have now been included.

Returning to the question raised above, the definition of impaired affect, based on direct questions about feelings of anxiety, nervousness, worry and depression, and the frequency of such feelings, seems inadequate to me.

RESPONSE:

Please see response to comment above: we have taken out reference to ‘impaired affect’ and instead use the terms ‘anxiety’ and ‘depression’. Further, we have also included more detailed reflections of the possible limitations of the approach we used to assess anxiety and depression in the limitations section of the discussion. [Page 25 Paragraph 2]

The study does not describe how exposure variables were measured.

RESPONSE:

We have now included more detail on these variables and the questions that were asked [Page 8 Paragraph 3]

Another methodological problem lies in the fact that there is no time definition for the onset of symptoms that led to the case definition. So how do we know about the exposure window for cases and controls? It would be correct to make a pairing of cases and controls, so that the onset date of the symptoms that classify the cases was taken to the time of exposure to the exposure variables also for the controls. In the current, nested case-control format, we have no way of knowing the temporality between a series of evaluated exposures and the outcome under study, such as marital status (for example, which came first, separation/divorce or mental disorder?), or even other variables. Such a format can lead to important biases.

RESPONSE:

Thank you for this comment. As you correctly state data were not collected on the onset of anxiety/depression symptoms or on date of exposures such as divorce/separation. We have now included this point in the limitations section, discussing the implications that it is not possible to establish causality/temporality of the associations observed.

The description of the study design (nested case-control) is insufficient. Include more details on eligibility criteria, etc. Were all non-cases included in the study?

RESPONSE:

The case control study included all people in the survey identified as having a disability (‘cases’) according to the study definition (i.e. reported a ‘lot of difficulty’ or ‘cannot do’ in at least one functional domain or ‘daily’ and ‘a lot’ to the anxiety/depression questions) and, for each case, one age-sex and cluster- matched control without a disability. Both cases and controls were interviewed about education, livelihoods and health. However, for the purposes of this paper we only analysed this data from the ‘cases’; comparing education, livelihood and health indicators between ‘cases’ with and without anxiety/depression to assess the additional burden of anxiety and/or depression.

We have now added more information on selection of case-control participants [Page 8 Paragraph 3] and we have edited the data analysis section [Page 10 Paragraph 3]

which we hope makes clearer who was included.

The inclusion of children – how was data collection conducted? There is no indication whether there has been an adaptation of the instrument, or whether it is suitable for children aged 5 years and over. Has any kind of assessment been made of the validity of questions about anxiety and depression symptoms for such young children? There is a straightforward question about depression. How does a child interpret this?
My suggestion is that the study only includes people aged 18 and over.

RESPONSE:

The CFM used in this study is an increasingly widely used tool recommended by UNICEF and the UN Washington Group on Disability statistics. We are therefore keen, if possible, to retain the information on children. However, we agree with the reviewers' concerns and have therefore discussed these issues in more detail and flagged the need for more research to validate these questions in the limitations section. We have also provided more details about the tool development and validation [Page 6 Paragraph 2] and explained that for children <11 years, parents were interviewed as proxy.

Results

The format for presenting the results is confusing. “Impaired affect” is the outcome under study; therefore, the tables should present the prevalence of the outcome according to the categories of the exposure variables. What the tables show is the frequency of exposures according to the outcomes, which is not adequate to meet the proposed objectives.

The results should include the crude and adjusted odds ratios.

RESPONSE:

As we are presenting the results of logistic regression analysis comparing characteristics of survey participants/’cases’ with and without ‘impaired affect’, we consider the way we have presented the data to be appropriate and are keen to keep that way if possible. We have now included unadjusted odds ratios as requested.

Discussion. The study has limitations that have been insufficiently discussed, namely: Diagnostic criteria for defining anxiety/depression:

The fact that WG-ES is based on self-report is an important limitation, but not the main one. In the case of the present study, not determining the onset of symptoms leads to problems that can lead to biases in the estimates found:

Temporality – there is no guarantee that some exposures occurred before the outcome.
Exposure windows for cases and controls may be different. If there was information about the date of onset of symptoms, there would be a possibility of pairing cases and controls, considering the exposure time in the paired control to be the same as in the case.

The comparison with the results found in other studies does not consider differences in these criteria, which can lead to differences in the prevalence of the outcomes under study.

RESPONSE:

Thank you for these comments. We have now expanded the limitations section in the discussion to discuss these issues in more detail. [Page 25 Paragraph 2]
