# Supplementary material for: Anxiety and depression in Guatemala: Sociodemographic characteristics and service access
Source: PLoS One. 2022 Aug 12;17(8):e0272780. doi: 10.1371/journal.pone.0272780 (PMC9374225; doi:10.1371/journal.pone.0272780)
Supplement: Attachment — Submitted filename: Response to Reviewers _ 20 June 2022.docx [file pone.0272780.s002.docx]

Dear Editor,

We thank you for the opportunity to resubmit our revised manuscript “Anxiety and depression in Guatemala: sociodemographic characteristics and service access”.

We also thank the reviewers for their thorough and helpful comments which we have responded to and which have strengthened the manuscript.

We have responded below to the ‘General Comments’ from PLOS ONE as well as to each of the reviewers and have made the revisions accordingly in the manuscript.

We look forward to hearing from you.

Yours faithfully,

Jonathan Naber

-----

**General comments: Journal requirements:**

1. Revise from “Cases (indicating ‘A lot’ or ‘Cannot do’ in 1+ function domain) and age-/sex-matched controls were administered a structured questionnaire” to “Cases (indicating ‘A lot of difficulty’ or ‘Cannot do’ in one or more functional domain) and age-/sex-matched controls were administered a structured questionnaire.” in the Abstract. (Page 2, Line 16)

RESPONSE:

We have made this change.

2. Delete “(WG-ES)” and “(CFM)” in the sentences “The WG ES was developed, tested, and adopted by the Washington Group on Disability Statistics ~~(WG-ES)~~” and “The CFM was developed and tested by the WG together with UNICEF ~~(CFM)~~.” (Page 6, Line 101-2)

RESPONSE:

We have made this change.

3. “We found no significant association between ethnic group and impaired affect in this study.” And additional 5-6 places having the term in in the text.

=> correct “impaired affect” into “anxiety and/or depression”

RESPONSE:

We have made these changes.

-----

**Reviewers’ Comments**

Reviewer #3: Excellently written article. Case control design and analysis are well designed and systematically carried out.

Tables are very clear.

Discussion refers to the references and findings.

Minor point: What is the reason to include the 5-17 group? (Table 3) The sample description in the table 2 showed 0-14 groups.

If authors are serious to add these age group, I think the logic to include them should be discussed.

Otherwise comparing children and old age may not make sense.

RESPONSE:

Table 2 compares the age and sex distribution of the study sample to that of the national population based on the most recent national census. We were restricted to using the 0-14 age group, for this comparison, because this is how the census data are presented by the Guatemalan National Institute of Statistics.

In table 3, we present findings for the 5-17 age group because the questions on symptoms of anxiety and depression were only asked to people aged 5+ years. Information on anxiety and/or depression among children in this setting is largely lacking from the literature. For example, the 2009 ENSM study completely omitted the under 18 age group and thus excluded a major part of the population. Therefore, we feel it is important to present this data, although the limitations of the measurement approach are acknowledged in the discussion.
